# Functional, Physical and Psychosocial Impacts of Oral Health-Related Quality of Life in Temporomandibular Disorders—A Comparative Study

**DOI:** 10.3390/diagnostics15050602

**Published:** 2025-03-02

**Authors:** Lujain AlSahman, Hamad AlBagieh, Roba AlSahman, Leopoldo P. Correa, Noshir R. Mehta

**Affiliations:** 1 Oral Medicine and Diagnostic Sciences Department, College of Dentistry, King Saud University Riyadh, Riyadh 57448, Saudi Arabia; 2 Faculty of Dentistry, Royal College of Surgeons, D02 YN77 Dublin, Ireland; 3 Campus Leon Guanajuato, Autonomous University of Mexico, C.P. 36000 Mexico City, Mexico; 4 Craniofacial Pain Center, Department of Diagnostic Sciences, Tufts University School of Dental Medicine, Boston, MA 02111, USA

**Keywords:** temporomandibular disorders, oral health-related quality of life, OHIP-TMD, diagnostic criteria for TMD, cross-sectional study

## Abstract

**Background/Objectives**: This study aims to evaluate the impact of temporomandibular disorders (TMDs) on oral health-related quality of life (OHRQoL), determine the effects of different influencing factors, and identify the most affected dimensions among the Saudi Arabian population. **Methods**: A cross-sectional study was conducted among 110 individuals visiting the Department of Oral Medicine at the Dental University Hospital (DUH), King Saud University. Participants were equally categorized into two groups: TMD and controls. The diagnosis was based on the Diagnostic Criteria for Temporomandibular Disorders (DC/TMDs). OHRQoL was assessed using the OHIP-TMD scale. Statistical analyses included independent *t*-tests, chi-square tests, and multivariate regression models to evaluate the association between TMD and OHRQoL. **Results**: The study population consisted of 72.7% females, with 91.8% holding a degree and 81.8% being married. TMD patients showed significantly lower OHRQoL scores in all domains (*p* ≤ 0.05), with the most pronounced impairments observed in terms of physical pain and psychological discomfort (*p* = 0.000). Marital status was a significant predictor of OHRQoL (*p* = 0.02; OR = 0.277), whereas gender and education showed no significant associations. **Conclusions**: TMD is significantly associated with impaired OHRQoL, particularly in the domains of physical pain and psychological discomfort. Marital status emerged as a significant demographic factor influencing OHRQoL. Given the cross-sectional nature of this study, the findings highlight associations rather than causation. Future longitudinal studies are recommended to establish causal relationships and further investigate the biopsychosocial impact of TMD on quality of life.

## 1. Introduction

Temporomandibular disorders (TMDs) encompass a diverse range of painful or dysfunctional conditions affecting the temporomandibular joints (TMJs), associated muscles, and surrounding structures [1,2]. The prevalence of TMD signs and symptoms varies greatly due to differences among populations [3]. The etiology of TMD follows a multifactorial biopsychosocial model, necessitating both physical and psychological management approaches. Effective evaluation of TMD should not only be based on clinical parameters but also patient-reported outcomes, including oral health-related quality of life (OHRQoL) [4]. TMD has been demonstrated to negatively affect everyday functioning and well-being, and it is highly prevalent in the young adult to middle-aged population [5,6,7]. The World Health Organization (WHO) defined oral health-related quality of life (OHRQoL) as the way in which an individual perceives oral health and its impact on their overall health, daily functioning, and quality of life [8]. Despite the growing body of research on temporomandibular disorders (TMDs) and oral health-related quality of life (OHRQoL), few studies have specifically assessed OHRQoL in TMD patients using the Diagnostic Criteria for Temporomandibular Disorders (DC/TMD). Moreover, demographic variables such as age, education, and marital status remain insufficiently studied. According to current prevalence rates, TMDs are significantly correlated with age, with the highest incidence occurring in individuals between the ages of 20–40 years [9].

The introduction of oral health-related quality of life (OHRQoL) has generated new perspectives for dental research, practice, and education by acknowledging the biopsychosocial effects of oral health and diseases on patients’ lives [10].

Numerous tools have been developed to measure the oral health-related quality of life (OHRQoL) among TMD patients. These include social indicators, global self-ratings, and multiple-item questionnaires, which have been extensively analyzed in TMD research [11,12,13]. Hence, a condition-specific and disease-sensitive OHIP-TMD scale was developed [13].

The primary OHIP-TMD, with 22 items, covers seven domains: functional limitation, physical pain, psychological discomfort, physical disability, psychological disability, social disability, and handicap. The OHIP-TMD 22 has demonstrated strong psychometric properties, including good test–retest reliability with an intraclass correlation coefficient of 0.805 (95% CI: 0.565, 0.918), as well as good face, content, and known groups validity. The items are scored on a five-point ordinal response scale ranging from “never” (0 points) to “very often” (4 points). Higher scores indicate poorer oral health-related quality of life. The OHIP-TMD 22 has been validated in clinical and non-clinical populations, making it a valuable tool for assessing the biopsychosocial impact of TMD on patients’ quality of life [14]. Many studies have compared OHIP-TMD with various other psychological and pain questionnaires without elucidating the strength of the association of each domain of OHIP-TMD among patients [11,15,16,17]. Moreover, the association between TMD experience and oral health-related quality of life (OHRQoL) remains underexplored among the adult population in Saudi Arabia. Given the region’s unique sociocultural and lifestyle factors, evaluating OHRQoL in this population offers novel insights into disease burden and patient-reported outcomes. Addressing this research gap is essential for developing tailored clinical strategies to improve the management and treatment of TMD in Saudi Arabia.

Therefore, we designed the present study to evaluate the oral health-related quality of life (OHRQoL) of the participants using the OHIP-TMD 22 scale. The objectives of the present study were to compare the OHRQoL of TMD adult patients to controls and determine the effects of demographic variables on the OHRQoL of patients with TMD.

Hence, the null hypotheses of the present study were as follows:

(a) No difference in OHRQoL in TMD patients and normal controls.
(b) Demographic variables do not influence the OHRQOL of TMD patients.
(c) All domains of OHIP-TMD will be equally affected in patients with TMD.

## 2. Materials and Methods

Participants

This cross-sectional study includes a total 110 participants, 30 males and 80 females, aged 18 to 40 years, who were enrolled in this cross-sectional study between January 2023 and March 2023 at Dental University Hospital (DUH), King Saud University, Saudi Arabia.

Selection criteria

The study subjects were divided into two groups: Group A (control group), comprising individuals without TMD, and Group B (study group), comprising patients with TMD. The inclusion criteria were symptomatic disc displacements (DDs) or/and myalgia according to the diagnostic criteria for temporomandibular joint disorders (DC/TMDs). Non-TMD individuals were those visiting the Dental University Hospital for routine dental treatment who matched the study group patients in age and gender.

The eligibility criteria for both the study and control groups included adult patients aged 18 to 40 years who were visiting the Dental University Hospital. Non-consenting patients, patients with conditions affecting pain sensitivity (e.g., fibromyalgia, rheumatoid arthritis, autoimmune diseases, migraines, or neurological and neuropsychiatric disorders), patients who had used anti-inflammatory drugs, opioids, analgesics, or steroids within the past 30 days (those who had discontinued use were eligible), and patients who were taking medications that affect saliva secretion (e.g., calcium channel blockers, antidepressants, antihistamines) were excluded. Additional exclusionary criteria included pregnancy or lactation, obesity, smoking, salivary gland diseases (e.g., tumors, stones, hyposalivation), complaints of dry mouth, edentulism, and prosthodontic rehabilitation (complete or partial dentures). Poor oral hygiene is defined by a Plaque Index (PI) or Gingival Index (GI) score above 2.0 and severe periodontal disease, according to the 2017 World Workshop on Periodontal and Peri-Implant classification (e.g., clinical attachment loss ≥ 5 mm, probing depths ≥ 6 mm, and significant bone loss). Mental health disorders that were untreated, being under active treatment for depression, anxiety, or PTSD, obstructive sleep apnea (OSA), malignancies, ongoing radiotherapy, ongoing chemotherapy, adrenal hyperfunction, and Cushing’s disease were further reasons for exclusion.

Sample size calculation

The sample size was calculated using the following formula:

n = (Z1 + Z2)2 × 2(S) 2/(μ2−μ1)2

where n = sample size for each group;

Z1 = 1.96, i.e., Z score for α error of 5% (95% confidence level);
Z2 = 0.84, i.e., Z score for estimated study power of 80%;
S = standard deviation 12.24 [18];
μ2 − μ1 = expected minimum difference between means of study and control groups = 7;

n = (1.96 + 0.84)2 × 2(12.24)2/(7)2 = (7.84) × 2(149.8)/49;

n = 48;

10% attrition rate = 5;

Participants per group = 53;

Total study participants = 106.

To account for an anticipated 10% attrition rate, the final calculated sample size per group was 53 participants, totaling 106 participants. However, 4 additional participants were included (total n = 110; 55 per group) to maximize data completeness and ensure adequate statistical power. This slight increase does not compromise the study’s validity and instead strengthens its robustness.

*Patient assessment*

TMD diagnosis was performed according to the Diagnostic Criteria for Temporomandibular disorder (DC/TMD) [19]. The patients maintained a sitting position with their heads positioned on the backrest of the chair while being examined clinically. A standardized form was used to record the demographic variables of the participating patients.

Clinical evaluation

This involved comprehensive assessment of the patient’s mandibular function and associated structures. Initially, the clinician measured the patient’s maximum unassisted and assisted mouth opening, as well as lateral and protrusive movements, to evaluate the range of motion and identify any limitations or deviations. During these movements, the temporomandibular joints were palpated to detect any joint sounds, such as clicking, popping, or crepitus, which may indicate internal derangements. Subsequently, gentle palpation of the masticatory muscles, including the temporalis and masseter, was performed to assess for tenderness or referred pain, providing insight into potential myofascial involvement. The TMJs were also palpated both during movement and at rest to identify any tenderness or pain, which could further inform the diagnosis.

*Oral health-related quality of life assessment*

All participants completed the OHIP-TMD questionnaire in the dental clinic. An examiner, blinded to the study protocol, assisted the participants who had difficulty in reading and writing. The instrument OHIP-TMD comprised twenty-two items and seven domains, i.e., functional limitation, physical pain, psychological discomfort, physical disability, psychological disability, social disability, and handicap. The participants’ responses were scored on a 5-point response scale varying from 0 = never to 4 = very often. Domain OHIP-TMD scores were obtained by totaling specified domains. The greater the scores, the poorer the OHRQoL will be [20,21].

*Statistical analysis*

Univariate analysis assessed demographic differences between groups. Descriptive statistics summarized OHIP-TMD domain-wise mean scores, followed by univariate analysis to compare item-wise responses between study and control groups. Multiple linear regression examined the relationship between TMD and oral health-related quality of life (OHRQOL) domains, with model fit assessed using R and R^2^ values. Scatter plots visualized linear relationships between significant domains and OHIP total scores. Multivariate analysis of variance (MANOVA) identified significant associations between domains and the outcome variable.

Data analysis was performed using IBM SPSS Statistics (version 23.0 for Windows; IBM Corporation, Armonk, NY, USA). Standard summary statistics were calculated for quantitative variables (number of patients, means, medians, and standard deviations) and qualitative variables (number of patients and percentages). Normality was confirmed using the Kolmogorov–Smirnov test. Intergroup comparisons were conducted using an independent *t*-test for quantitative variables and a chi-square test for qualitative variables.

Multivariate regression identified OHIP-TMD domains significantly affecting TMD patients in this population. Statistical significance was set at *p* < 0.05.

## 3. Results

A total of 110 patients participated in this study. The majority (72.7%) were female, while 27.3% were male. Most participants were degree holders (91.8%) and married (81.8%). Univariate analysis of demographic variables revealed statistically significant differences between the control and TMD groups (Table 1).

**Figure 1:**
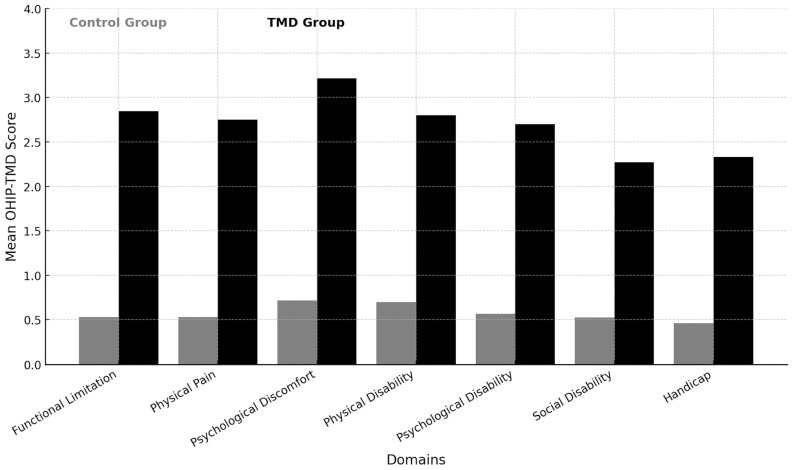
Domain-wise mean scores for the control versus study group.

Table 2 presents the mean OHIP-TMD domain-wise descriptive statistics for each group. The overall mean item-wise score was higher in Group B than in Group A. The domain-wise mean scores for the control versus study group were as follows: functional limitation (0.53 ± 0.99 vs. 2.84 ± 1.63), physical pain (0.52 ± 0.97 vs. 2.75 ± 1.55), psychological discomfort (0.71 ± 1.16 vs. 3.21 ± 1.35), physical disability (0.70 ± 1.16 vs. 2.80 ± 1.74), psychological disability (0.56 ± 1.04 vs. 2.70 ± 1.59), social disability (0.52 ± 1.04 vs. 2.27 ± 1.68), and handicap (0.46 ± 1.00 vs. 2.33 ± 1.86) (Figure 1). Univariate analysis of ordinal-scale responses showed a statistically significant difference between the study and control groups (*p* ≤ 0.05), with higher mean values in the study group.

In multiple logistic regression (Table 3), for predicting the oral health-related quality of life in TMD participants as measured by the OHIP-TMD scale, only marital status (*p* = 0.02; OR = 0.277) had a significant effect, while gender and education showed no significant effects (*p* > 0.05).

Multiple linear regression analysis (Table 4) indicated that TMD patients had significantly lower OHRQOL in the physical pain and psychological discomfort domains.

The model summary indicates a strong relationship between OHIP domains and TMD. The multiple correlation coefficient (R) is 0.812, reflecting a strong positive correlation. The R Square value of 0.659 shows that 65.9% of TMD variance is explained by OHIP domains, with the remaining 34.1% attributable to other factors.

The Adjusted R Square value is 0.608 (60.8%), which is slightly lower than the R Square value. With a value above 0.60, it reflects strong model fit and a conservative estimate of explanatory power. The Standard Error of the Estimate is 0.016, indicating high predictive precision.

Examining the individual OHIP domains, psychological discomfort (B = 0.047, *p* < 0.001) and physical pain (B = 0.031, *p* = 0.004) are the only statistically significant predictors. These domains have the strongest positive associations with TMD, while other domains show minimal effects and are not statistically significant (*p* > 0.05). This model suggests that the OHIP domains collectively explain a substantial portion (65.9%) of TMD variation. Among them, psychological discomfort and physical pain are the most important predictors. The model also demonstrates good predictive accuracy due to the low standard error, and the high R value confirms strong overall predictive power.

The multiple linear regression model predicting OHIP total scores in the TMD group included all seven OHIP-TMD domains based on clinical relevance and prior research. Multicollinearity was assessed, and predictors with weak associations (*p* > 0.05) were considered for removal unless clinically justified. Potential interactions, such as physical pain × psychological discomfort and physical disability × psychological disability, were explored, but only physical pain and psychological discomfort remained significant.

**Figure 2:**
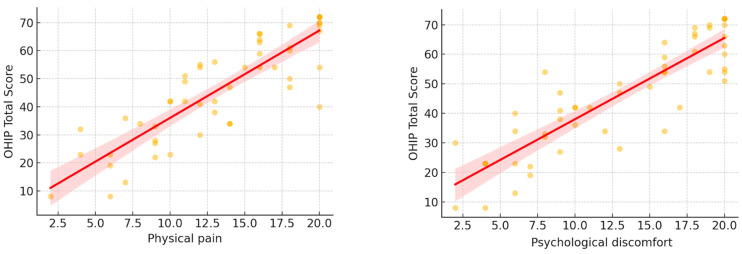
Plots of the significant domains. The red shadow: regression line, the yellow dots: observed values.

Figure 2 presents scatter plots of the significant domains, demonstrating strong linear relationships between physical pain, psychological discomfort, and OHIP total scores in TMD patients.

Multivariate analysis of variance (MANOVA) (Table 5) identified significant associations across several domains: functional limitation (β = 0.35, *p* < 0.001), physical pain (β = 0.42, *p* < 0.001), and psychological discomfort (β = 0.39, *p* < 0.001) had strong positive relationships with the outcome variable, indicating that higher scores in these domains were significantly associated with worse OHRQOL. Psychological disability also showed a significant positive association (β = 0.25, *p* = 0.002), confirming its contribution to the model.

## 4. Discussion

In this study, we aimed to compare the OHRQoL between individuals with temporomandibular disorders and those without, using the TMD-specific Oral Health Impact Profile (OHIP-TMD) questionnaire, specifically within the Saudi Arabian population. The present study is the first of its kind in the Saudi Arabian population.

The first and third null hypotheses were rejected as there was a significant difference in OHRQOL between the study and control groups. This finding highlights the degree of negative impact of TMD on the patients’ OHRQOL. The second null hypothesis cannot be accepted as marital status was significantly associated with OHRQoL, while age and gender were not. The inclusion of young adults and middle-aged participants was intentional, as these age groups align with the peak manifestation period of TMD [7,17]. Our findings indicate that TMD participants were affected across all seven domains measured by OHIP-22, with the most considerable effects detected in psychological discomfort, functional limitation, and physical pain. The mean OHIP-TMD scores were higher for TMD patients than for normal controls, demonstrating that OHIP-TMD is a sensitive and valid condition-specific scale for the present population in delineating TMD. Therefore, OHIP-TMD can be used as an effective self-reported adjunct tool for TMD. The study’s main outcome showed that TMD negatively affected oral health-related quality of life (OHRQoL) in every domain evaluated by the OHIP-22. This indicates that TMD has a wide-ranging impact on patients’ perceived well-being, consistent with other studies that identified a link between TMD and lower overall OHRQoL [22,23].

While participants in the study were found to have higher scores in all seven domains compared to normal controls, the regression analysis indicated that both physical pain and psychological discomfort had a significant impact on OHRQoL (B = 0.031, *p* = 0.004 and B = 0.047, *p* < 0.001, respectively). These two domains were the only statistically significant predictors, reinforcing their critical role in TMD-related OHRQoL impairment. These findings are consistent with previous research, which has shown that physical pain and psychological distress are the most affected domains in TMD patients [16,17,24,25,26].

This helps to explain why treatments such as Non-Steroidal Anti-Inflammatory Drugs (NSAIDs) for pain management and selective serotonin reuptake inhibitors (SSRIs) for alleviating psychological discomfort are effective in improving the OHRQoL of TMD patients, as they target these crucial factors [27]. In this cross-sectional study, the study group included participants with symptomatic TMD disc displacements (DDs) and myalgia, which is consistent with previous findings indicating that painful TMD—primarily muscular and joint pain—is strongly associated with poorer OHRQoL [28,29].

Painful TMD is associated with lower OHRQoL, underscoring the significant impact of these conditions on patients’ overall quality of life. Therefore, it is important to periodically assess patients’ OHRQoL as part of managing painful TMD.

Polonowita et al. highlighted the importance of moving away from a “one size fits all” approach to a more personalized, patient-centered care model for managing TMD. They found that when TMD treatment was complemented with simple self-help strategies, it significantly enhanced the sense of coherence among TMD patients, leading to improvements in their overall quality of life. This suggests that a more individualized and comprehensive treatment approach is essential for effectively managing TMD and improving patients’ quality of life [30].

This study contributes to the field by highlighting the negative relationship between TMD and OHRQoL domains, as well as overall scores, using a representative sample of the Saudi population. The decision to use the specific OHIP-TMD scale over generic scales limited comparisons with previous TMD studies that used different measurement tools. However, given the increasing prevalence of TMD, it is essential to adopt comprehensive, condition-specific measurement tools to develop effective preventive and curative strategies for this fast-emerging public health concern.

Limitations of the study: the age group range in this study limited the population size which may have reduced the power of this study since some patients with TMD outside the study age were not captured.

## 5. Conclusions

Temporomandibular disorder (TMD) patients showed significantly lower oral health-related quality of life (OHRQoL), particularly in the domains of physical pain and psychological discomfort. The findings indicate an association between TMD and compromised OHRQoL, with marital status emerging as a significant demographic factor related to OHRQoL. Given the cross-sectional nature of the study, these results highlight associations rather than causal relationships. Future longitudinal studies are necessary to establish causal links and further explore the biopsychosocial impact of TMD on quality of life. We are of the opinion that evaluation using OHIP-TMD in the management of TMD will be very beneficial.

Recommendation: Dental professionals should be encouraged to assess TMD when evaluating patients’ overall oral health status and its impact on daily functioning.

Moreover, the psychosocial discomfort domain is as significantly affected as physical pain in TMD patients. Therefore, curative therapy should not only target symptoms but also incorporate interdisciplinary approaches that include psychosocial support for long-term management. Clinicians should integrate psychosocial support and counseling to enhance and implement tailored pain management strategies that address both physical and emotional distress in TMD patients. Finally, patient education on the biopsychosocial aspects of TMD should be emphasized to improve self-management and adherence to treatment plans.

## Figures and Tables

**Table 1:** Demographics and chi-square test between groups.

Category	Overall (*N,* %)	Control Group (*N*, %)	TMD Group (*N*, %)	Chi-Square *p*-Value
Gender				
Females	80 (72.7%)	40 (72.7%)	40 (72.7%)	Not significant
Males	30 (27.3%)	15 (27.3%)	15 (27.3%)	
Age				Not significant
18–30	49 (44.5%)	26 (47.3%)	23 (41.8%)	
31–40	61 (55.5%)	29 (52.7%)	32 (58.2%)	
Education				
Degree	101 (91.8%)	48 (87.3%)	53 (96.4%)	0.000 *
High School	9 (8.2%)	7 (12.7%)	2 (3.6%)	
Marital Status				
Married	90 (81.8%)	40 (72.7%)	50 (90.9%)	0.001 *
Single	20 (18.2%)	15 (27.3%)	5 (9.1%)	

* Statistically significant at *p* < 0.05.

**Table 2:** Domain-wise mean scores and univariate analysis of OHIP –TMD control and TMD group at 95% CI.

Domain	Overall Mean + SD	Overall with a 95% CI	Control Group Mean + SD	TMD Group Mean + SD	T	*p*-Value
Functional limitation	1.68 + 1.78	1.69 [95% CI: 1.35–2.02]	0.53 + 0.99	2.84 + 1.63	8.8625	0.000 *
Physical pain	1.63 + 1.72	1.64 [95% CI: 1.31–1.96]	0.52 + 0.97	2.75 + 1.55	8.8292	0.000 *
Psychological discomfort	1.96 + 1.80	1.97 [95% CI: 1.62–2.31]	0.71 + 1.16	3.21 + 1.35	6.896	0.000 *
Physical disability	1.75 + 1.82	1.75 [95% CI: 1.41–2.09]	0.70 + 1.164	2.80 + 1.74	6.896	0.000 *
Psychological disability	1.63 + 1.74	1.63 [95% CI: 1.3–1.96]	0.56 + 1.04	2.70 + 1.59	8.0398	0.000 *
Social disability	1.41 + 1.65	1.4 [95% CI: 1.09–1.71]	0.52 + 1.04	2.27 + 1.68	6.1955	0.000 *
Handicap	1.43 + 1.76	1.4 [95% CI: 1.07–1.73]	0.46 + 1.00	2.33 + 1.86	6.1255	0.000 *

*—statistically significant. T—independent ‘T’ test. CI = confidence intervals.

**Table 3:** Logistic regression for gender, education, and marital status at 95% CI.

	TMD Group (55 Participants)		
	Standard Error	Odds Ratio	*p* Value
Gender	0.450	0.867	0.752
Education	0.851	0.285	0.140
Marital status	0.259	0.277	0.024 *

*—statistically significant.

**Table 4:** Multiple linear regression for predicting the effect of OHIP in the TMD group at 95% CI.

Domain	Mean TMD	B	Standard Error	*p*-Value
Functional Limitation	4.88	0.002	0.01	0.920
Physical Pain	12.90	0.031	0.01	0.004 *
Psychological Discomfort	11.86	0.047	0.01	0.001 *
Physical Disability	5.58	−0.012	0.01	0.396
Psychological Disability	11.20	−0.006	0.01	0.594
Social Disability	4.53	−0.003	0.02	0.874
Handicap	4.15	−0.003	0.01	0.870
**Model Summary:**				
R	0.812			
R Square	0.659			
Adjusted R Square	0.608			
Std. Error of the Estimate	0.016			

*—statistically significant.

**Table 5:** Multivariate analysis of variance (MANOVA) results for OHIP domains comparing TMD and control groups.

Domains	B	Standard Error	β (Beta)	t-Value	*p*-Value
Functional Limitation	0.25	0.05	0.35	5.00	0.001 *
Physical Pain	0.40	0.07	0.42	5.71	0.001 *
Psychological Discomfort	0.38	0.06	0.39	6.33	0.001 *
Physical Disability	0.15	0.08	0.18	1.88	0.064
Psychological Disability	0.22	0.07	0.25	3.14	0.002 *
Social Disability	0.10	0.09	0.12	1.11	0.269
Handicap	0.18	0.08	0.20	2.25	0.027

*—statistically significant.

## Data Availability

The datasets used and/or analyzed during the current study are available from the corresponding author upon reasonable request.

## Abbreviations

TMDs	Temporomandibular disorders
OHRQoL	Oral health-related quality of life
OHIP-TMDs	Oral health impact profile for temporomandibular disorders
DC/TMDs	Diagnostic Criteria for Temporomandibular Disorders
TMJ(s)	Temporomandibular joint(s)
DDs	Disc displacements
OSA	Obstructive sleep apnea
PTSD	Post-Traumatic Stress Disorder
NSAIDs	Non-Steroidal Anti-Inflammatory Drugs
SSRIs	Selective Serotonin Reuptake Inhibitors
PI	Plaque Index
GI	Gingival Index
IRB	Institutional Review Board
SPSS	Statistical Package for the Social Sciences (IBM SPSS Statistics)
CI	Confidence interval
OR	Odds ratio
